# Steroid Response after Trabeculectomy—A Randomized Controlled Trial Comparing Dexamethasone to Diclofenac Eye Drops

**DOI:** 10.3390/jcm11247365

**Published:** 2022-12-12

**Authors:** Afrouz Ahmadzadeh, Line Kessel, Bo Simmendefeldt Schmidt, Daniella Bach-Holm

**Affiliations:** 1 Department of Ophthalmology, Copenhagen University Hospital—Rigshospitalet, 2600 Copenhagen, Denmark; 2 Department of Clinical Medicine, University of Copenhagen, 2100 Copenhagen, Denmark; 3 Department of Physics, Technical University of Denmark, 2800 Kongens Lyngby, Denmark

**Keywords:** glaucoma, NSAID, steroid, trabeculectomy

## Abstract

This prospective randomized controlled trial aimed to compare changes in intraocular pressure in three different anti-inflammatory regimens following trabeculectomy. Sixty-nine patients were randomized to receive either postoperative prophylaxis with topical preservative-free dexamethasone (DEX), diclofenac (DICLO), or their combination (DEX+DICLO). Our main outcome measure was an intraocular pressure (IOP) change of a minimum 4 mmHg following the withdrawal of anti-inflammatory prophylaxis 9 weeks after trabeculectomy. We found that the IOP decreased ≥ 4 mmHg in 18.6% of eyes after cessation of the topical steroid DEX (n = 3/22) and DEX+DICLO (n = 5/21), whereas a decrease in IOP was not observed in the DICLO group. In conclusion, IOP decreased in nearly 1/5 of patients after cessation of topical steroidal anti-inflammatory prophylaxis after trabeculectomy. This points toward a steroid-induced increase in IOP even after trabeculectomy. Thus, increased postoperative IOP may be related to steroid use, and the success or failure of a trabeculectomy cannot be fully evaluated before anti-inflammatory prophylaxis with steroids is stopped or changed to non-steroidal eye drops.

## 1. Introduction

Increased intraocular pressure (IOP) is a well-documented adverse effect of steroids [1]. Steroid responders are individuals who are susceptible to increased IOP during systemic or topical steroid therapy. This side effect of corticosteroids is more prevalent in glaucoma patients and children than in the general population [2,3,4,5]. The mechanism by which steroids cause elevated IOP is not fully known, but morphological changes in the trabecular meshwork associated with increased resistance of aqueous outflow are considered a possible site of action [6]. 

The treatment of choice for medically uncontrolled glaucoma is trabeculectomy, as elevated IOP remains the major modifiable risk factor for preserving visual function [7]. To control the inflammation, postoperative topical anti-inflammatory medication is used, most commonly steroids [8,9]. In a successful trabeculectomy, the aqueous humour leaves the eye through the bleb; therefore, steroid-induced changes in the trabecular meshwork should have minimal or no influence on IOP. However, it has been observed that some patients have elevated IOP during topical treatment with steroids after a trabeculectomy despite a functional filtering bleb [10,11]. This may lead to reoperations, such as needling or a revision of the trabeculectomy, due to the impression that the primary surgery was ineffective. 

Non-steroidal anti-inflammatory drugs (NSAID) are attractive alternatives as postoperative treatment after a trabeculectomy, as they have not been associated with IOP increase. Additionally, previous studies have shown that NSAIDs are comparable with topical steroids in controlling early inflammation after trabeculectomy and cataract surgery [12,13,14].

In this randomized controlled clinical trial, we sought to identify if corticosteroid use is associated with a rise in IOP following trabeculectomy.

## 2. Materials and Methods

The Steroids and/or Non-steroidal Anti-inflammatory Drugs in the Postoperative Regime After Trabeculectomy Study (SNAP) is a prospective randomized controlled clinical trial conducted at the Department of Ophthalmology at Rigshospitalet-Glostrup, Denmark. Three topical and preservative-free anti-inflammatory regimens, dexamethasone (DEX) (Monopex 1 mg/mL, Théa), diclofenac (DICLO) (Voltaren Ophtha 1 mg/mL, GSK Consumer Healthcare) or a combination of dexamethasone (Monopex 1 mg/mL, Théa) and diclofenac (Voltaren Ophtha 1 mg/mL, GSK Consumer Healthcare) (DEX+DICLO) following trabeculectomy were compared. 

A topical antibiotic (Chloramphenicol 5 mg/mL) was prescribed four times daily for the first week. Anti-inflammatory prophylaxis was planned to last a minimum nine weeks. For the first two weeks, the drops were used six times daily, followed by four drops daily for four weeks. After six weeks, the topical anti-inflammatory medication was reduced by one daily drop per week, depending on the clinical status of the eye. Preservative-free topical DEX was used if topical therapy was required for more than 15 weeks following the filtration operation.

The study was approved by the Danish Committee on Health Research Ethics (Journal nr.: H-18056701), the Danish Medicines Agency (Journal nr.: 2018082465) and the Danish Data Protection Agency (VD-2018-477, I-Suite nr.: 6736). The study was registered at www.clinicaltrials.gov (accessed on 7 December 2022) (NCT04054830) and the European Union Drug Regulating Authorities Clinical Trials Database (EudraCT, 2018-001855-10), was conducted in accordance with Good Clinical Practice guidelines, and adhered to the tenets of the Declaration of Helsinki [15]. All participants provided written informed consent and received no incentives or compensation for participation. The Consolidated Standards of Reporting Trials (CONSORT) reporting guideline was followed.

### 2.1. Study Participants

Participants were recruited from 1 August 2019 to 11 July 2021, from patients scheduled for trabeculectomy at the Department of Ophthalmology, Rigshospitalet-Glostrup, Denmark. Participants had to be older than 50 years, and women had to be postmenopausal. We included participants with either primary open-angle glaucoma (POAG), pseudoexfoliation syndrome (PEX), pigment dispersion syndrome (PDS) or ocular hypertension (OH). Participants had to be able to comply with study procedures and consent to participation.

Exclusion criteria were known allergy to any content of the pharmaceuticals used in the study, previous history of steroid response mentioned in the medical charts (using the definition of the treating physician or confirmed by questioning study participants), systemic treatment with NSAIDs or steroids, prior intraocular surgery (except for cataract surgery), medical history of anterior segment dysgenesis, inflammatory/uveitic glaucoma, angle closure glaucoma, neovascular glaucoma or traumatic glaucoma. 

### 2.2. Randomization and Blinding

**Figure 1:**
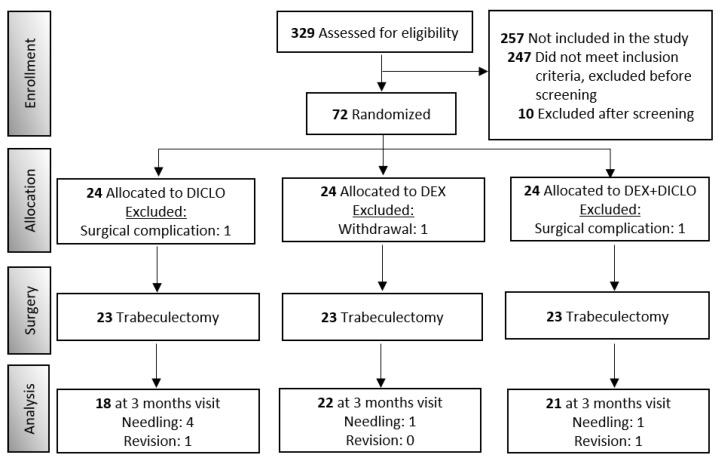
Consort Diagram for IOP analysis.

Participants were randomized to one of the three interventional groups (Figure 1). A computerized algorithm determined which eye to include in the study if both eyes were eligible. An independent researcher generated a block-randomized list in Sealed Envelope (https://www.sealedenvelope.com/simple-randomiser/v1/lists (accessed on 7 December 2022)) and uploaded it to Research Electronic Data Capture (REDCap) [16,17], a randomization instrument hosted at Capital Region, Denmark. The list length was 123, with block sizes 6 and 9 in random order. The study was designed as single blinded, with primary outcome assessors masked to the randomization status. All statistical calculations were performed in a blinded manner.

### 2.3. Surgical Technique

The trabeculectomies were performed by three experienced surgeons with a standard limbus-based scleral technique using 0.2 mg/mL Mitomycin (MMC) on sponges subconjunctivally for 3 min. The surgery was concluded by injecting 1 mL cefuroxime 2.5 mg/mL into the anterior chamber and applying 0.5 mL of 4 mg/mL dexamethasone 180° away from the trabeculectomy subconjunctivally. One operation was performed using general anesthesia, and all other surgical procedures were performed using peribulbar or topical anesthesia according to the surgeon’s preference. 

### 2.4. Follow-Up Examinations and Outcome

The primary outcome in the present study was the IOP change from 6 weeks to 3 months postoperatively. IOP was measured using Goldmann applanation tonometry. Two measurements were taken and averaged to the mean IOP if the two values were within 2 mmHg. A third measurement was taken if the first two deviated ≥3 mmHg. If so, the median value was used [18]. Participants were identified as steroid responders if they experienced a pressure decrease ≥4 mmHg at 3 months (off topical anti-inflammatory eye drops) compared to 6 weeks after surgery (on anti-inflammatory eye drops). At 6 weeks, patients received 4 or 8 anti-inflammatory eye drops a day (according to the randomization), whereas participants had been free of anti-inflammatory eye drops for 4 weeks at the 3 month visit. If a participant required surgical intervention, e.g., needling or revision, in the first 3 postoperative months, the patient was excluded from the analysis due to the incomparable postoperative medical prophylaxis with steroids.

### 2.5. Statistical Analysis 

A one-way ANOVA was used to calculate *p*-values for the baseline characteristics of the participants, including gender, age, pre-operative IOP, MD and the number of glaucoma medications; see Table 1. The dependent variables were tested for normality using Shapiro–Wilk’s test and for homogeneity of variances using Levene’s test. From the IOP changes from 6 to 12 weeks, participants who experienced a drop in IOP (steroid responders) were analyzed using descriptive statistics for inter-group differences. 

## 3. Results

Between August 2019 and June 2021, 72 participants were randomized and scheduled for trabeculectomy. One participant withdrew consent, two experienced complications during surgery, six required needling, and two required revision. A total of 61 eyes (27 women (44%) and 34 men (56%)) were included in the analyses with a mean age of 71.5 ± 8.8 years (range, 51 to 88 years). Figure 1 and Table 1 summarize the demographics of the study sample. 

A reduction ≥4 mmHg was observed in 18.6% of eyes that received steroids (n = 8/43). None of the participants in the DICLO group had a reduction ≥4 mmHg following cessation of anti-inflammatory prophylaxis. After discontinuation of anti-inflammatory prophylaxis, 8.2% (n = 5/61) of study participants had unchanged IOP, whereas IOP increased in 32.8% (n = 20/61). For those participants who experienced an increase after prophylactic treatment stopped, the mean IOP reached 11.3 mmHg (6.8 to 15.7 mmHg, 95% CI) in the DICLO group, compared to 9.5 mmHg (8.2 to 10.8 mmHg, 95% CI, DEX group) and 12.4 mmHg (10.5 to 14.3 mmHg, 95% CI, DEX+DICLO group) in the steroid groups. The mean IOP at 3 months was 8.8 mmHg for all participants with no significant difference between groups; see Table 2.

## 4. Discussion

We investigated if an anti-inflammatory regime after trabeculectomy affects IOP by evaluating the change in IOP after cessation of anti-inflammatory prophylaxis. We used a cut-off of a 4 mmHg decrease in IOP from 6 weeks (during anti-inflammatory prophylaxis) to 3 months (4 weeks after withdrawal of anti-inflammatory prophylaxis). We found that nearly one in five subjects receiving topical dexamethasone (18.6 %, n = 8/43) experienced a decrease in IOP after cessation of anti-inflammatory prophylaxis, whereas such a reduction was not observed among those who received non-steroidal anti-inflammatory prophylaxis alone (n = 0/18). This observation indicates that there is a risk of a steroid-induced increase in intraocular pressure after trabeculectomy. Notably, patients who were known to be steroid responders were excluded from the study. Topical steroids (compared to no anti-inflammatory prophylaxis) generally improves outcomes after trabeculectomy [19,20]; however, our results suggest that apparent surgical failure (increased IOP) during steroid therapy may be related to steroid use and that a steroid-free period may be warranted before deciding if needling or a revision of the trabeculectomy should be performed. 

The definition of steroid responders in the general population varies among studies. Yamamoto et al. [21] and Abtahi et al. [22] define steroid responders as individuals with an IOP rise of 5 mmHg from baseline, whereas Chang et al. describe steroid responsiveness as an IOP increase of at least 25% when on topical prednisolone, and up to 28 mmHg, followed by a drop of at least 25%, when the medication is withdrawn [23]. Steroid response may be divided into three categories: approximately 2/3 are low responders, with a pressure increase <6 mmHg and an IOP < 20 mmHg; approximately 1/3 are intermediate responders, with IOPs between 20 and 31 mmHg or a pressure increase of 6–15 mmHg; and 4–6% are high responders, with an IOP above 31 mm Hg or an increase of more than 15 mm Hg above baseline [24]. Typically, these steroid responders are young, or have a family history of glaucoma, myopia or diabetes [2,3,4,5]. 

When using topical steroids, IOP increases three to six weeks after initiation and usually returns to normal within two weeks after discontinuation [25]. Considering that our participants had undergone a trabeculectomy (and there should have been no aqueous outflow resistance at the trabecular meshwork), we set our steroid response cut-off at 4 mmHg. Although we excluded known steroid responders, we found that 18.6% of participants experienced a decrease in IOP of ≥4 mmHg after cessation of topical steroids, whereas this was not observed in the group that did not receive steroids. 

Reports describing the occurrence of steroid response after trabeculectomy are limited. Thomas et al. studied 87 eyes of 52 participants with POAG and found a significant steroid-induced increase in 23% of the eyes with a mean pressure of 25.1 mmHg; Wilensky et al. reported an IOP between 25 to 42 mmHg following trabeculectomy during postoperative steroid prophylaxis [10,11]. The incidence of steroid response after combined phacoemulsification with either Trabectome or iStent is reported to be 12.7% [22]. Several studies have assessed the efficacy of steroids and NSAIDs in controlling early postoperative inflammation. Regarding anterior chamber flare, no significant difference was found among the different anti-inflammatory treatments [26,27,28]. Taking the inflammation parameter into account, no participant in the DICLO group had an IOP decrease ≥ 4 mmHg, compared to eight participants in the DEX and DEX+DICLO groups following their sessions of anti-inflammatory treatment. Surgical manipulation leading to increased inflammation may have contributed to this transient postoperative increase in IOP. To ascertain this, histological testing of the trabecular meshwork would be necessary. 

NSAID eye drops may be a good alternative to topical steroids after filtering surgery as they have an anti-inflammatory effect without an IOP increase during treatment. The most common complications associated with topical NSAID are irritation when the eye drop is applied, impaired vision shortly after application, redness and corneal melts that usually occur in individuals with a cornea weakened due to diabetes, ocular surgery or systemic immunological disorders, and have only been documented in rare cases [29,30].

The key strengths of our trial were its randomized design, large sample size and a control group that did not receive steroids. As one group had combination therapy, the study could not be fully masked. However, the primary outcome assessors and all statistical analyses were conducted in a blinded manner. Increased IOP was observed in 1/3 of participants between the three-month and six-week visits, likely due to bleb fibrosis. It would have been informative to compare bleb appearance between treatment groups to ascertain the effect of the previously administered anti-inflammatory prophylaxis.

## 5. Conclusions

Nearly 1/5 of individuals undergoing trabeculectomy experienced increased intraocular pressure during postoperative treatment with topical steroids—an increase that was not seen after NSAID treatment. It is important to recognize a steroid response following trabeculectomy since erroneous decisions may be taken, such as initiating a prolonged pressure lowering treatment or intervening surgically. Steroid treatment should be discontinued before determining if a trabeculectomy is unsuccessful and further measures should be taken. 

## Figures and Tables

**Table 1:** Baseline characteristics.

	All Participants	DICLO	DEX	DEX + DICLO	*p*-Value
Participants, n	61	18	22	21	
F/M, n (%)	27/34 (44/56)	5/13 (27.8/72.2)	12/10 (54.5/45.5)	10/11 (47.6/52.4)	0.40
Age (yrs), mean (SD)	71.5 (8.8)	70.1 (7.3)	73.2 (8.0)	71.0 (10.8)	0.53
Pre-operative IOP (mm Hg), mean (SD)	18.8 (5.8)	18.2 (6.8)	18.4 (5.7)	19.8 (5.2)	0.62
Visual field MD (dB), mean (SD)	15.3 (5.7)	16.0 (5.4)	15.1 (6.6)	15.0 (5.1)	0.86
No. of glaucoma medications, mean (SD)	3.4 (0.8)	3.5 (0.8)	3.3 (0.8)	3.5 (0.7)	0.64
* Prostaglandin, n (%)*	61 (100)	18 (100)	22 (100)	21 (100)	
* Beta-blocker, n (%)*	56 (91.8)	16 (88.9)	20 (90.9)	20 (95.2)	
* Topical Carbonic Anhydrase Inhibitor, n (%)*	56 (91.8)	17 (94.4)	19 (86.4)	20 (95.2)	
* Alpha agonist, n (%)*	37 (60.7)	12 (66.7)	12 (54.5)	13 (61.9)	
* Systemic Carbonic Anhydrase Inhibitor, n (%)*	7 (11.5)	2 (11.1)	2 (9.1)	3 (14.3)	

F/M = female/male; SD = standard deviation; IOP = intraocular pressure; MD = mean deviation; HTG = high tension glaucoma; NTG = normal tension glaucoma; PXG = pseudoexfoliative glaucoma; PG = pigmentary glaucoma; OH = ocular hypertension.

**Table 2:** Change in IOP from 6 weeks to 3 months.

	All Participants	DICLO	DEX	DEX+DICLO	STEROID (DEX, DEX + DICLO)
Participants, n	61	18	22	21	43
IOP at 6 w, (mmHg), mean (SD)	9.4 (3.7)	8.6 (3.3)	9.1 (4.0)	10.3 (3.7)	9.7 (3.8)
IOP at 3 m, (mmHg), mean (SD)	8.8 (2.61)	8.6 (2.6)	9.0 (3.3)	8.9 (2.0)	8.9 (2.7)
Increased IOP, n (%)	20 (32.8)	7 (38.9)	6 (27.3)	7 (33.3)	13 (30.2)
Increased IOP, (mmHg), mean (SD) *	11.1 (2.9)	11.3 (4.2)	9.5 (1.2)	12.4 (2.1)	11.1 (2.3)
Unchanged IOP, n (%)	5 (8.2)	2 (11.1)	1 (4.5)	2 (9.5)	3 (7.0)
Decreased IOP, n (%)					
0 to < −2 mmHg	14 (23.0)	3 (16.7)	8 (36.4)	3 (14.3)	11 (25.6)
−2 to < −4 mmHg	14 (23.0)	6 (33.3)	4 (18.2)	4 (19.0)	8 (18.6)
−4 to < −6 mmHg	3 (4.9)	-	2 (9.1)	1 (4.8)	3 (7.0)
−6 to < −8 mmHg	4 (6.6)	-	1 † (4.5)	3 (14.3)	4 (9.3)
−8 to < −10 mmHg	1 (1.6)	-	-	1 † (4.8)	1 (2.3)

Table 2 shows changes in IOP from when patients were using topical eye drops (6 weeks) to when topical eye drops use stopped (3 months). Thus, higher pressure during eye drop treatment was seen as a drop in IOP at 3 months. IOP = intraocular pressure (mean value); w = week; m = month; * IOP indicates participants who experienced increased pressure after discontinuation of anti-inflammatory prophylaxis; † indicates two participants who were suspected to be anti-inflammatory responders and received topical anti-glaucomatous treatment.

## Data Availability

The datasets generated and/or analyzed during this study are available from the corresponding author upon reasonable request.
